# Tiny Trouble, Unknown Risk: International Interceptions Highlight Cross-Border Movement and Biosecurity Threat of *Cenopalpus* (Acari: Tenuipalpidae)

**DOI:** 10.3390/insects17030290

**Published:** 2026-03-06

**Authors:** Marcello De Giosa, Sauro Simoni, Tobias E. Glik, Michael Ormsby, Helen Nahrung, Aline D. Tassi, Ronald Ochoa, Davina L. Saccaggi

**Affiliations:** 1 Tropical Research and Education Center, Department of Entomology and Nematology, University of Florida, Homestead, FL 33031, USA; aline.tassi@ufl.edu; 2 Council for Agricultural Research and Economics, Research Center for Plant Protection and Certification, 50125 Florence, Italy; sauro.simoni@crea.gov.it; 3 Plant Protection and Quarantine, USDA Animal and Plant Health Inspection Service, Romulus, MI 48174, USA; tobias.e.glik@usda.gov; 4 Ministry for Primary Industries, Wellington P.O. Box 25266140, New Zealand; michael.ormsby@mpi.govt.nz; 5 Forest Research Institute, University of the Sunshine Coast, Sippy Downs, QLD 4556, Australia; hnahrung@usc.edu.au; 6 Systematic Entomology Laboratory, Agricultural Research Service-United States Department of Agriculture, Beltsville Agricultural Research Center-West, Beltsville, MD 20705, USA; ron.ochoa@usda.gov; 7 Citrus Research International (CRI), Stellenbosch 7602, South Africa; davinas@cri.co.za; 8 Department of Conservation Ecology and Entomology, Stellenbosch University, Stellenbosch 7602, South Africa

**Keywords:** flat mites, quarantine, pest, interceptions, invasions, dispersal

## Abstract

Mite species of the genus *Cenopalpus* are phytophagous pests associated with fruit and ornamental crops. Although the genus is primarily distributed in the Palearctic region, increasing international trade of plant material has created repeated opportunities for its unintentional transport across borders. To support improved awareness of emerging biosecurity risks, international quarantine interception records were analyzed from Australia, Laos, Malaysia, New Zealand, Singapore, South Africa, and the United States of America. A total of 98 interceptions were documented between 1981 and 2024, exclusively reported by New Zealand, South Africa, and the USA, while no *Cenopalpus* spp. mites were intercepted in Australia, Laos, Malaysia, or Singapore. Six species were identified, with *Cenopalpus pulcher* being the most frequently detected, followed by *C. lanceolatisetae* and *C. officinalis*. Interceptions were primarily associated with fruit crops, particularly apples and stone fruits, frequently with origins in Europe and the Middle East. Several records represent the first detection of *Cenopalpus* in trade originating from countries within Africa, Asia, the Americas, and Oceania. Taxonomic uncertainty associated with *Cenopalpus* mites likely limits accurate identification in interception records, highlighting the need for improved diagnostic expertise. The implications of these findings for plant biosecurity programs, including surveillance and diagnostic priorities, are discussed.

## Simple Summary

Mites of the genus *Cenopalpus* are plant-feeding pests that can cause significant damage to fruit and ornamental plants, such as apples and peaches. These mites are mainly found in Asia, Europe, and northern Africa, but the global trade of plants is facilitating their spread to new areas. This study examined official border inspections from several countries to understand how often and where *Cenopalpus* mites are being transported. Between 1981 and 2024, a total of 98 interceptions were recorded in New Zealand, South Africa, and the USA, while no interceptions were reported from Australia, Laos, Malaysia, or Singapore. The most frequently detected species were the flat scarlet mite, *Cenopalpus pulcher*, followed by *C*. *lanceolatisetae*. Most interceptions involved fruit plants, especially apples and stone fruits, imported from Europe and the Middle East. Some of these reports represent first interception records in trade, providing evidence of cross-border movement that may pose a growing threat to agriculture. Taxonomic uncertainty may make it difficult to identify *Cenopalpus* mites in interception records, showing the need for better diagnostic expertise. These findings highlight the importance of continued surveillance and accurate identification of mites in plant trade to support plant biosecurity and prevent the establishment of new pests.

## 1. Introduction

The family Tenuipalpidae Berlese comprises 41 genera of plant-feeding mites, many of which are recognized as agricultural pests due to their ability to cause crop damage and transmit plant pathogens, compromising the productivity and aesthetic quality of the crop [1,2]. Tenuipalpid mites are globally distributed, with the highest number of genera described from Australia and Africa [3]. The largest and most widespread genera are *Tenuipalpus* Donnadieu and *Brevipalpus* Donnadieu, with 333 and 290 valid species, respectively, comprising more than a third of described tenuipalpid species [4,5]. In contrast, *Cenopalpus* Pritchard & Baker, the third largest genus, contains only 74 valid species, concentrated primarily in Asia, where 54 species have been recorded, followed by 17 species in Europe and three in northern African countries along the Mediterranean coast [4,5,6].

Historically, *Cenopalpus* has received limited attention relative to *Tenuipalpus* and *Brevipalpus*, partly because no species within this genus are known to act as vectors of plant pathogens, and because severe crop damage has rarely been reported [2,7,8]. *Cenopalpus* occurs primarily in the Palearctic region (Asia, Europe, and North Africa), where it exhibits comparatively lower biodiversity and a more restricted distribution, with only sparse records reported outside this region [4,5]. In recent years, increasing relevance has been attributed to this genus in the context of plant biosecurity, as multiple species have been detected outside their historically recognized ranges [9,10,11,12,13]. Since, in many cases, the economic impact of *Cenopalpus* species is unknown, the spread of different species through commerce represents an unknown risk factor. These detections suggest that international movement of plant material may create repeated opportunities for the unintentional transport of *Cenopalpus* species across borders.

This concern aligns with broader global trends, as the rapid expansion of international trade and the commercialization of agricultural products have facilitated the spread of species beyond their native ranges [14,15]. Small-bodied organisms, such as mites, are particularly prone to transport via plant products due to their cryptic habits and limited detectability during inspections [16,17]. This is also true for *Cenopalpus,* as detections of this genus have been increasingly noted in internationally traded fruit, herb, and plant propagation material [11,18,19]. For instance, *Cenopalpus pulcher* (Canestrini et Fanzago), a polyphagous species with a preference for Rosaceae hosts such as apples (*Malus* spp.), has expanded from Asia and Europe to the Americas, with early records in Argentina [20] and later in the USA [9]. This species was most likely introduced as a contaminant on infested plants or plant material [9]. Similarly, *Cenopalpus officinalis* Papaioannou-Souliotis, which primarily infests rosemary (*Rosmarinus officinalis* L., Lamiaceae), a Mediterranean-native now widely cultivated worldwide, has recently been reported in Mexico [10,11,21,22]. These recent introductions of *Cenopalpus* species raise fundamental concerns about the ability to recognize and quarantine these pests worldwide.

The two most widespread species, *Cenopalpus lanceolatisetae* (Attiah) and *C. pulcher*, are already on the quarantine lists for several countries (variously termed quarantine, regulated, or prohibited pests). *Cenopalpus lanceolatisetae* is a quarantine pest for the USA, New Zealand, and South Africa, while *C. pulcher* is a quarantine pest for Puerto Rico and Hawaii, New Zealand, South Africa, Brazil, and Morocco [23,24,25,26]. Further, *Cenopalpus irani* Dosse has recently been categorized by the European Food Safety Authority (EFSA) as a potential threat to the European Union, although it is not yet listed as a regulated pest [27]. The economic impact of other species is largely unknown, and they are not regulated. To mitigate these biosecurity risks, countries have developed protocols aimed at preventing the introduction and spread of invasive organisms and diseases [17]. In most cases, efforts include updating inspection protocols for imported plant products and recording any contaminant organisms detected. These records are primarily intended to inform biosecurity decision-making and implement rapid responses to quarantine threats [17,28]. Records can also be used to reveal species movement patterns and plant associations, analyze historical trends, determine introduction risk, and develop targeted risk-reduction protocols [18,29,30,31]. However, there are important limitations to the interpretation of biosecurity data, and misinterpretation can have serious economic consequences [17,18]. Interception data are collected for biosecurity decision-making, and they are biased towards economically destructive pests not present in the country. Uncommon or unknown species or taxa are less likely to be detected, identified, or recorded in interception records, particularly if these are small or cryptic [17,30]. Furthermore, unresolved taxonomy often results in incomplete or incorrect identifications, especially when compounded by a lack of taxonomic experts in plant protection agencies [32]. This is particularly true for *Cenopalpus*, a lesser-known genus that can easily be misidentified as the more common *Brevipalpus*.

With these limitations in mind, the present study analyzes quarantine interception records of *Cenopalpus* species from seven countries across multiple continents to characterize patterns of international transport, host associations, and geographic origins. By analyzing historical interception data spanning more than 4 decades, we aim to (i) identify the *Cenopalpus* species most frequently transported in trade, (ii) determine the plant commodities most commonly associated with interceptions, and (iii) highlight regions that may be repeatedly exposed to this genus through international plant movement.

Knowledge of which species are frequently intercepted can be used to adjust risk-based biosecurity protocols [18] and make inferences regarding the risk of entry and establishment in new regions [33]. Frequently transported and intercepted species are more likely to be introduced and established in new areas [34,35]. Improved knowledge of the prevalence of *Cenopalpus* in international trade will assist in biosecurity decisions and hopefully encourage additional research regarding this genus.

## 2. Materials and Methods

### 2.1. Data Acquisition, Limitations, and Handling

The authors approached colleagues working in biosecurity in various countries to enquire about *Cenopalpus* interceptions. Because interception records were obtained from different national databases, the inspection effort, metadata, level of detail, and temporal coverage varied among countries. Where available, metadata such as country of origin, plant host, type of commodity, year of interception, and final taxonomic identification were recorded. We were able to obtain interception data from the following seven countries:

South Africa. Interception data was extracted from published information, originally collated from the Department of Agriculture, Plant Health Diagnostic Services, Stellenbosch [23]. The dataset used spans 15 years, from 1994 to 2019. For these samples, inspection was performed using a stereomicroscope, and both positive and negative results were recorded. A total of 185 tenuipalpid interceptions were recorded, of which 44 were *Cenopalpus* [23].

United States of America. Interception data was obtained directly from the United States Department of Agriculture, Animal and Plant Health Inspection Service and Plant Protection and Quarantine (USDA-APHIS-PPQ). The dataset spans 24 years, from 1981 to 2024. The inspection method was not specified, although it was typically performed with a stereomicroscope when mites were found (RO, personal observation), and only positive results were recorded. Fifty *Cenopalpus* interceptions were recorded, but total tenuipalpid interceptions were not specified.

Australia. Interception data was obtained directly from the Department of Agriculture, Fisheries and Forestry (DAFF). The dataset spans 10 years, from 2015 to 2024. Inspection practices varied according to the commodity and targeted pest. Where mites were detected, a stereomicroscope inspection was performed. A total of 534 tenuipalpid interceptions were recorded, with no *Cenopalpus* interceptions.

New Zealand. Interception data was obtained directly from the Ministry for Primary Industries. The records span 27 years, from 1998 to 2025. The inspection methodology was not specified, and only positive results were recorded. Many tenuipalpid interceptions were recorded (over 100), but most of these were identified to genus only. Only four *Cenopalpus* interceptions were recorded, all from a single instance of confiscation of fresh produce from air passenger luggage in 2010.

Malaysia. Information was obtained from the Plant Biosecurity Division. Records are available for 19 years, from 2006 to 2025. Methods of sampling and inspection were not specified, and only positive results were recorded. In total, 283 mite interceptions were recorded, but the number of tenuipalpids was not specified, and no *Cenopalpus* interceptions were recorded.

Lao People’s Democratic Republic (Laos). Information was obtained from the Plant Quarantine Division, Ministry of Agriculture and Forestry (MAF). No details regarding time frame, inspection methods, or recording of data were divulged. No *Cenopalpus* interceptions were recorded.

Singapore. Information was obtained from the Animal & Plant Health Centre. No details regarding time frame, inspection methods, or recording of data were divulged. No *Cenopalpus* interceptions were recorded.

Only the data from South Africa, the USA, and Australia were of sufficient detail to allow analysis. Information on inspection effort, total inspection volume, and negative records was not consistently available across all countries. Consequently, comparisons between countries in this study should be considered descriptive rather than quantitative assessments of relative risk. The absence of *Cenopalpus* interceptions in some countries may reflect the available datasets and should not be interpreted as evidence of total absence in imported commodities. Interceptions, or lack thereof, from other countries are presented, but not included in analyses.

All records were compiled into a standardized Microsoft Excel database for further analysis.

### 2.2. Taxonomic Identification and Data Validation

Identifications of intercepted specimens were conducted by taxonomists affiliated with the respective quarantine agencies or collaborating research institutions, and no specimens were re-examined for this study. Identification was based on adult morphology using available taxonomic keys. To minimize inconsistencies, all scientific names were verified against the most recent version of the Tenuipalpidae Database [5], and outdated synonyms were updated when necessary. Records in which specimens were identified only to genus level were retained in the dataset but treated separately from species-level analyses.

### 2.3. Trade Data

Summary trade data was obtained from World Integrated Trade Solutions [36], using “vegetable imports” as the search term. In this context, “vegetable imports” covers all plant material and edible fresh plant products imports, such as propagation material, fruit, and vegetables. It does not cover processed plant products such as dried, canned, or manufactured goods.

Graphs were generated using the ggplot2 and gridExtra packages in RStudio within the R statistical computing environment [37,38,39,40].

## 3. Results

**Figure 1:**
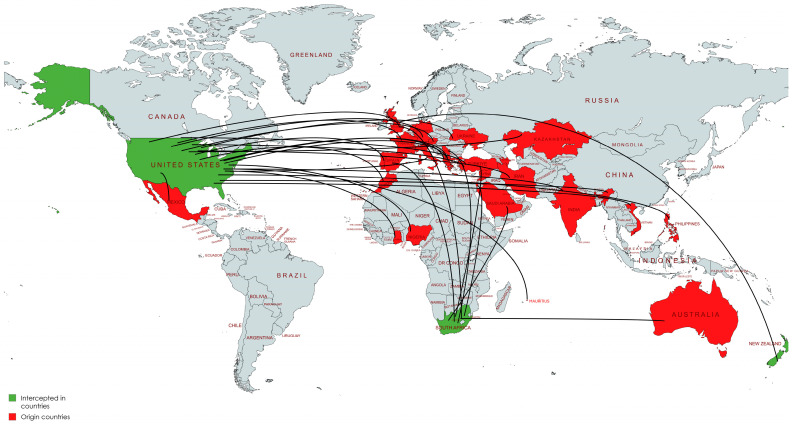
Global interception pathways of *Cenopalpus* mites from 1981–2024. Importing countries where interceptions were recorded are shown in green, while exporting countries representing the origins of infested plant material are shown in red. Curved flow lines indicate movement pathways linking source countries to interception destinations.

A total of 98 interceptions of six *Cenopalpus* species were recorded from only three countries (USA, South Africa, and New Zealand) in the examined datasets (Figure 1). Most commonly intercepted were *Cenopalpus pulcher* (52 interceptions), followed by *Cenopalpus lanceolatisetae* (26 interceptions) and *Cenopalpus officinalis* (16 interceptions). *Cenopalpus* mites were often detected on fruit crops, especially apple, most commonly on propagation material but sometimes on fresh fruit (when this was recorded).

### Interceptions by Country, Species, Host, and Origin

**Figure 2:**
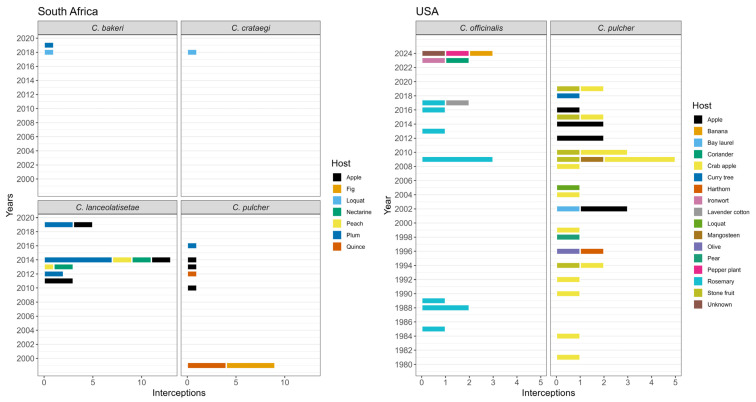
Annual interception records of *Cenopalpus* species in South Africa and the United States, categorized by host plant. Each bar represents the number of interceptions associated with a specific host in a given year. The temporal analysis includes data only from these two countries.

The USA recorded the most *Cenopalpus* interceptions, with 50 interceptions recorded between 1981 and 2024 (Figure 2). However, yearly interceptions of *Cenopalpus* were fairly low, with an average of 1.14 interceptions per year (maximum of eight) and 17 years having no interceptions at all (Figure 2). Only two species were intercepted: *C. pulcher* and *C. officinalis*. Of the 50 interceptions, 34 (68%) were *C. pulcher*, predominantly found on fruit crops, including 22 on apple and crab apple (*Malus domestica* and other *Malus* spp.), and 5 on stone fruit (*Prunus* spp.), with the remaining 7 on other fruit crops. The remaining 16 interceptions (32%) were *C. officinalis*, mostly intercepted on rosemary (*Rosmarinus officinalis*), its primary host, with other interceptions on culinary or ornamental plants. Commodity type was recorded in only 15 of the interceptions. Both mite species were commonly intercepted from European countries, including the United Kingdom, with 71% of *C. pulcher* and 44% of *C. officinalis* interceptions originating from this region. Additional interceptions of *C. pulcher* were recorded from Africa, Asia, the Middle East, and Serbia, and of *C. officinalis* from Africa, Asia, the Middle East, and South America.

South Africa recorded the second-highest number of *Cenopalpus* interceptions and the highest diversity of species, with 47 interceptions and five species intercepted between 1999 and 2019 (Figure 2). The average number of interceptions per year was 1.96 interceptions, with the highest number recorded in 2014 (14 interceptions) and 11 years with no interceptions. The most common species intercepted by South Africa was *C. lanceolatisetae* (26 interceptions, 59%) and *C. pulcher* (14 interceptions, 32%). Two interceptions of *C. bakeri*, and one each of *Cenopalpus spinosus* (Donnadieu) and *Cenopalpus crataegi* Dosse were also recorded. All South African interceptions were on stone fruit (*Prunus* spp.) (19 interceptions). *Cenopalpus pulcher* was intercepted primarily on apple (*Malus domestica*), fig (*Ficus carica*) and quince (*Cydonia oblonga*). The South African records all included the commodity type, and the majority of interceptions were on propagation material (41 interceptions). In contrast to the USA, most interceptions were from the Middle East, with the majority of *C. lanceolatisetae* interceptions from Israel (96%). Additional interceptions were recorded from Africa, Australia, and Europe (including the United Kingdom).

New Zealand only recorded four interceptions in 2010 of *C. pulcher* on fresh apples confiscated from travelers, two from the United Kingdom and two from an unknown origin.

No interceptions of *Cenopalpus* species were recorded in Australia, Laos, Malaysia, and Singapore.

## 4. Discussion

International trade in plant products facilitates the movement of arthropods beyond their native ranges, sometimes resulting in economic and ecological impacts of considerable magnitude [41]. Despite the knowledge that mites can be serious agricultural pests, work on adventive mites or mites transported in trade is still lacking [42,43]. This is especially true for minor genera, such as *Cenopalpus*, with few associated researchers and uncertain economic transports. Our results demonstrate that *Cenopalpus* is recurrently transported via international commerce and should be regarded as an emerging biosecurity concern.

### 4.1. Differences in Country Interceptions

The three countries with the most detailed datasets, namely the USA, South Africa, and Australia, record different numbers and species of *Cenopalpus* intercepted. Other countries report only a few (New Zealand) or none (Laos, Malaysia and Singapore), but their datasets are lacking in detail. This could be due to any number of reasons.

Patterns of interceptions have been shown to be closely related to how frequently and in what volumes host plants or plant products are imported from countries where *Cenopalpus* is present [18,34]. Between 1992 and 2023, the USA imported an average of 42.46 million US dollars of plant and vegetable products per year, Australia 2.14 million US dollars, and South Africa 1.88 million US dollars [36]. In the interception datasets, the USA reported an average of 1.14 interceptions per year, Australia none, and South Africa 2.2. Clearly, the average number of interceptions is not related to overall import volumes.

The differences may rather be due to different trading partners, the specific plant hosts or types of commodities imported, or sampling and inspection strategies targeting high-risk imports. However, this is difficult to determine due to the limited nature of biosecurity interception information, where details of sampling and inspection protocols (including negative results) are seldom recorded [17]. In some cases, the absence may reflect stricter biosecurity measures and improved sanitation of plants prior to shipment [44]. Even if mites are present in imports, several factors influence whether they are detected. These include biosecurity approaches and priorities of a country, which may change from time to time [17,45]. Mites are difficult to detect during quarantine inspections, requiring specialized equipment and trained personnel. If certain species are not a quarantine priority, then the extra time, labor, and money allocated to detection and identification may not be justified. Even for countries in which mite detections are a priority, low numbers of mites present in the commodity may not be detectable given realistic sampling protocols [30,46].

For Australia, a high number of *Brevipalpus* interceptions were made over the same period, which indicates that the commodities imported and their associated biosecurity protocols are likely to transport and detect tenuipalpid mites. Therefore, we can assume that in this case, the commodities imported are truly free of *Cenopalpus*, although whether due to trading patterns or stricter biosecurity is unknown.

One can speculate on possible reasons for the differences, but without detailed long-term data, it is not possible to accurately determine which factors influence transport and interception of mites on a global scale [17,18,30,31,35].

### 4.2. Species Interceptions and Taxonomic Bias

Among the 74 described *Cenopalpus* species [5,6], only 6 species were present in the examined datasets, and only *C. pulcher* was common to all three countries, with positive interception records. The predominance of only three species (*C. pulcher*, *C. lanceolatisetae* and *C. officinalis*) may reflect taxonomic uncertainty rather than true abundance. Adult tenuipalpids, including *Cenopalpus*, are difficult to identify because many cryptic species share nearly identical dorsal and ventral reticulation patterns, and nymphs are critical for species-level identification [32]. Adults of *C. lanceolatisetae*, *C. bakeri*, *C. crataegi*, and *C. pulcher* closely resemble each other, and since interceptions frequently involve very few adult specimens and no nymphs, accurate identification is complicated [17]. Given these circumstances, plant quarantine officers may prefer to only identify to family or genus level, or may tend to match specimens to known quarantine species, such as *C. lanceolatisetae* in South Africa or *C. pulcher* in the USA and New Zealand, which could explain their reported frequency.

### 4.3. Increased Distribution of Cenopalpus Species

Among the intercepted species, *C. pulcher* was the most commonly intercepted species. It is also the species known to have spread to multiple world regions [5]. The success of *C. pulcher* in spreading to new regions can be attributed to several factors: its polyphagous feeding behavior, which allows it to survive and develop large populations on multiple host plants [1], the global popularity of apples, and the mass propagation practices of *Malus* species that facilitate its movement between countries. New *Malus* varieties are commonly introduced to new regions as dormant budwood and propagated by grafting. Mites migrate to the dormant buds for the winter, where they lay their eggs [1]. Both adult mites and eggs can be easily overlooked at ports of entry due to their small size and hidden location within the buds [17]. Eggs may then hatch under favorable environmental conditions in new regions, as occurred in Oregon, USA, in 2001, where *C. pulcher* is now well established in apple orchards [9,47]. The predominance of this species in the interception records reflects its propensity to be transported on plant products and may be an early predictor of introductions to areas where it is not yet found [31,33].

Despite the large numbers of interceptions of *C. lanceolatisetae* in South Africa and the extensive cultivation of its preferred host, *Prunus* spp. (stone fruit), this species has not established in South Africa, as far as is known. This may be attributable to South Africa’s strict post-entry quarantine system, which imposes prolonged observation of imported propagation material [23]. Although causality cannot be directly demonstrated, this system likely reduces establishment probability by allowing latent infestations to be detected and eliminated before commercial distribution.

Interceptions of *Cenopalpus* species that feed on herbaceous medicinal plants, such as *C. officinalis* on herbs, are expected to rise due to the growing significance of these plants in health and wellness, personal care, hygiene products [48,49], as well as gastronomy tourism, which may involve the personal cross-border transport by travelers [50].

*Cenopalpus pulcher* and *C. officinalis* have been intercepted from countries within the Western Palearctic and Oriental regions. Although these countries fall within their native ranges, these represent the first trade interception records of the genus in the United Arab Emirates, North Macedonia, the Philippines, Serbia, and Vietnam. Beyond the genus’s expected native range, *C. pulcher* and *C. officinalis* have also been intercepted from Ghana and Nigeria, and other *Cenopalpus* specimens, not identified to the species level, have been intercepted from Australia, Cameroon, Colombia, and the Dominican Republic, also representing the first trade records of the genus in these countries. Prior to this study, in the Neotropical region, *Cenopalpus* had been reported only from Argentina [20], Mexico [51], and Peru [12]. The new records from the Dominican Republic and Colombia increase the total to five countries, extending the known distribution of the genus in the Neotropical region. Similarly, within the Afrotropical region, four species had been reported from Southern Africa, specifically South Africa [52,53], one from Eastern Africa, including Mauritius [13], and 14 species from North Africa, including Algeria, Egypt, Libya, Morocco, and Tunisia [5]. Previously, no species had been reported from Central or West Africa. The current records from Cameroon, Ghana, and Nigeria are therefore from countries where *Cenopalpus* was not previously known. Furthermore, *Cenopalpus* species have now been intercepted from the Australian region, including New Zealand and Australia, which were previously considered uncolonized by this genus [4]. However, caution is needed when interpreting these records: an interception does not necessarily indicate a new country or the establishment of the species, as imported plants can transit through multiple countries before reaching their final destination. For example, in the Netherlands, plants arriving at ports of entry from around the world may be redistributed to other locations, where they can become infested with mites [19]. Similarly, transient or accidental transport on non-hosts may occur. For example, a single specimen of *C*. *officinalis* was detected on a leaf of *Musa* sp., a plant on which it does not typically feed. *Cenopalpus officinalis* prefers herbs rather than tree fruits [51], suggesting that *Musa* is likely not a true host, and the specimen was potentially a result of contamination during transportation. Therefore, further field surveys and plant samplings are necessary to confirm the presence of *Cenopalpus* in these areas.

## 5. Conclusions

The patterns of interceptions presented in this paper give a glimpse into the occurrence of *Cenopalpus* on commonly traded plant products. With increasing global trade, transport of contaminant organisms is likely to increase, also increasing the probability of establishment in new regions. This is highlighted by our findings that many *Cenopalpus* interceptions are from regions where they have not previously been recorded as present. The presence of *Cenopalpus* in additional regions further increases the chances of transport on traded plant products, as each additional region acts as an additional source.

Although *Cenopalpus* is a relatively minor genus compared with *Brevipalpus* and *Tenuipalpus*, its movement has increased considerably during the 21st century. The pest status of many *Cenopalpus* species remains poorly understood and may have greater impacts than previously appreciated. Their frequent occurrence in international trade highlights the need for further research on this understudied genus.

## Data Availability

The original contributions presented in this study are included in the article. Further inquiries can be directed to the corresponding author.

## References

[1] Jeppson L.R., Keifer H.H., Baker E.W. (1975). Mites Injurious to Economic Plants.

[2] de Lillo E., Freitas-Astúa J., Kitajima E.W., Ramos-González P.L., Simoni S., Tassi A.D., Valenzano D. (2021). Phytophagous mites transmitting plant viruses: Update and perspectives. Entomol. Gen..

[3] Castro E.B., Mesa N.C., Feres R.J., De Moraes G.J., Ochoa R., Beard J.J., Demite P.R. (2020). A newly available database of an important family of phytophagous mites: Tenuipalpidae Database. Zootaxa.

[4] Mesa N.C., Ochoa R., Welbourn W.C., Evans G.A., De Moraes G.J. (2009). A catalog of the Tenuipalpidae (Acari) of the World with a key to genera. Zootaxa.

[5] Castro E.B., Mesa N.C., Feres R.J.F., de Moraes G.J., Ochoa R., Beard J.J., Demite P.R. (2026). Tenuipalpidae Database. http://www.tenuipalpidae.ibilce.unesp.br.

[6] De Giosa M., Tassi A.D., Varela A.R., Silva M.J., Nóbrega F., Jansen A.M., Ochoa R., Naves P. (2025). A comprehensive study on *Cenopalpus* (Acari: Tenuipalpidae) species in Portugal: Morphological, molecular, and annotated review. Syst. Appl. Acarol..

[7] Nizi G. (1963). Lotta contro l’acaro dei pini *Cenopalpus lineola*. Note Appunti Entomol. Agr..

[8] Elmosa H.M. (1971). Studies on the Biology of *Cenopalpus pulcher* (C. and F.) (Acarina, Tenuipalpidae) in Baghdad. Z. Angew. Entomol..

[9] Bajwa W.I., Krantz G.W., Kogan M. (2001). Discovery of *Cenopalpus pulcher* (C. & F.) (Acari: Tenuipalpidae) in the New World. Int. J. Acarol..

[10] Flamini G., Cioni P.L., Morelli I., Macchia M., Ceccarini L. (2002). Main agronomic− productive characteristics of two ecotypes of *Rosmarinus officinalis* L. and chemical composition of their essential oils. J. Agric. Food Chem..

[11] De Giosa M., Bassini-Silva R., de Lillo E., McDonald E.M., Ochoa R. (2021). Italian Acarine species intercepted in the United States. Int. J. Acarol..

[12] Huanca J., De Giosa M., Bauchan G., Evans G., Ochoa R. (2022). First Record of *Cenopalpus wainsteini* [Trombidiformes: Tetranychoidea: Tenuipalpidae] in the Americas and a description of the symptoms it causes on pines in Peru. Neotrop. Entomol..

[13] De Giosa M., Ochoa R., Castro E.B., Simoni S., Glik T., Tassi A.D., de Lillo E. (2024). Updated Italian checklist of Tenuipalpidae with description of a new species and new worldwide records of the genus *Cenopalpus* (Pritchard et Baker). Int. J. Acarol..

[14] Liebhold A.M., Brockerhoff E.G., Garrett L.J., Parke J.L., Britton K.O. (2012). Live plant imports: The major pathway for forest insect and pathogen invasions of the US. Front. Ecol. Environ..

[15] Faulkner K.T., Robertson M.P., Rouget M., Wilson J.R. (2016). Border control for stowaway alien species should be prioritised based on variations in establishment debt. J. Environ. Manag..

[16] Navia D., Ochoa R., Welbourn C., Ferragut F. (2010). Adventive eriophyoid mites: A global review of their impact, pathways, prevention and challenges. Exp. Appl. Acarol..

[17] Saccaggi D.L., Karsten M., Robertson M.P., Kumschick S., Somers M.J., Wilson J.R., Terblanche J.S. (2016). Methods and approaches for the management of arthropod border incursions. Biol. Invasions.

[18] Saccaggi D.L., Wilson J.R., Robinson A.P., Terblanche J.S. (2022). Arthropods on imported plant products: Volumes predict general trends while contextual details enhance predictive power. Ecol. Appl..

[19] Zhovnerchuk O.V., Ochoa R., Glik T.E., Tassi A.D., De Giosa M. (2025). Distribution advances of Tenuipalpidae in Central and Eastern Europe. Acarologia.

[20] Proley C.F., Villanueva G., Amadio L. Control de *Cenopalpus* sp. (Acari: Tenuipalpidae) en el Alto Valle de Rio Negro y Neuquen [Argentina, *Malus domestica*, oxythioquinox, flubenzamin, clorobencilato, propargite, dimetoato, control de insectos, plaguicidas]. Proceedings of the 5th Jornadas Fitosanitarias Argentinas.

[21] González-Minero F.J., Bravo-Díaz L., Ayala-Gómez A. (2020). *Rosmarinus officinalis* L. (Rosemary): An ancient plant with uses in personal healthcare and cosmetics. Cosmetics.

[22] Aziz E., Batool R., Akhtar W., Shahzad T., Malik A., Shah M.A., Iqbal S., Rauf A., Zengin G., Bouyahya A. (2022). Rosemary species: A review of phytochemicals, bioactivities and industrial applications. S. Afr. J. Bot..

[23] Saccaggi D.L., Arendse M., Wilson J.R., Terblanche J.S. (2021). Contaminant organisms recorded on plant product imports to South Africa 1994–2019. Sci. Data.

[24] APHIS 2025 U.S. Regulated Plant Pest Table. https://www.aphis.usda.gov/plant-imports/regulated-pest-list.

[25] EPPO Global Database. https://gd.eppo.int.

[26] MPI Official New Zealand Pest Register. https://pierpestregister.mpi.govt.nz/.

[27] Bragard C., Baptista P., Chatzivassiliou E., Di Serio F., Gonthier P., Jaques Miret J.A., Justesen A.F., Magnusson C.S., Milonas P., EFSA Panel on Plant Health (PLH) (2024). Pest categorisation of *Cenopalpus irani*. EFSA J..

[28] McCullough D.G., Work T.T., Cavey J.F., Liebhold A.M., Marshall D. (2006). Interceptions of nonindigenous plant pests at US ports of entry and border crossings over a 17-year period. Biol. Invasions.

[29] Roques A., Auger-Rozenberg M.A. (2006). Tentative analysis of the interceptions of non-indigenous organisms in Europe during 1995–2004. EPPO Bull..

[30] Turner R.M., Plank M.J., Brockerhoff E.G., Pawson S., Liebhold A.M., James A. (2020). Considering Unseen Arrivals in Predictions of Establishment Risk Based on Border Biosecurity Interceptions. Ecol. Appl..

[31] Turner R.M., Brockerhoff E.G., Bertelsmeier C., Blake R.E., Caton B., James A., MacLeod A., Nahrung H.F., Pawson S.M., Plank M.J. (2021). Worldwide Border Interceptions Provide a Window into Human-Mediated Global Insect Movement. Ecol. Appl..

[32] Saccaggi D.L., Ueckermann E.A. (2024). The problem of taxonomic uncertainty in biosecurity: South African mite interceptions as an example. Acarologia.

[33] Caley P., Ingram R., De Barro P. (2015). Entry of exotic insects into Australia: Does border interception count match incursion risk?. Biol. Invasions.

[34] Brockerhoff E.G., Kimberley M., Liebhold A.M., Haack R.A., Cavey J.F. (2014). Predicting how altering propagule pressure changes establishment rates of biological invaders across species pools. Ecology.

[35] Turner R.M., Liebhold A.M., Nahrung H.F., Phillips C.B., Yamanaka T., Brockerhoff E.G. (2025). The known unknowns in international border interceptions of non-native insects. Biol. Invasions.

[36] WITS (World Integrated Trade Solutions). https://wits.worldbank.org.

[37] Auguie B. gridExtra: Miscellaneous Functions for “Grid” Graphics. R Package Version 2.3. https://CRAN.R-project.org/package=gridExtra.

[38] Wickham H. (2016). ggplot2: Elegant Graphics for Data Analysis.

[39] R Core Team R: A Language and Environment for Statistical Computing.

[40] Posit Team RStudio: Integrated Development Environment for R. Posit Software, PBC, Boston, MA. http://www.posit.co/.

[41] Lopes-da-Silva M., Sanches M.M., Stancioli A.R., Alves G., Sugayama R. (2014). The role of natural and human-mediated pathways for invasive agricultural pests: A historical analysis of cases from Brazil. Agric. Sci..

[42] Pyšek P., Richardson D.M., Pergl J., Jarošík V., Sixtová Z., Weber E. (2008). Geographical and taxonomic biases in invasion ecology. Trends Ecol. Evol..

[43] Navajas M., Ochoa R. (2013). Integrating ecology and genetics to address Acari invasions. Exp. Appl. Acarol..

[44] Whattam M., Azzopardi S., Nehl D., Maxwell A., Davis K. (2024). Protecting Australia’s plant health: Plant quarantine in an evolving biosecurity system. Hist. Rec. Aust. Sci..

[45] Eschen R., Britton K., Brockerhoff E., Burgess T., Dalley V., Epanchin-Niell R.S., Gupta K., Hardy G., Huang Y., Kenis M. (2015). International variation in phytosanitary legislation and regulations governing importation of plants for planting. Environ. Sci. Policy.

[46] Venette R.C., Moon R.D., Hutchison W.D. (2002). Strategies and statistics of sampling for rare individuals. Annu. Rev. Entomol..

[47] Rodrigues J.C.V., Gallo-Meagher M., Ochoa R., Childers C.C., Adams B.J. (2004). Mitochondrial DNA and RAPD polymorphisms in the haploid mite *Brevipalpus phoenicis* (Acari: Tenuipalpidae). Exp. Appl. Acarol..

[48] Nastić L., Jeločnik M., Subić J. (2024). Economic efficiency of investments in the growing of medicinal herbs and spices. Econ. Agric..

[49] Pergola M., De Falco E., Belliggiano A., Ievoli C. (2024). The Most Relevant Socio-Economic Aspects of Medicinal and Aromatic Plants through a Literature Review. Agriculture.

[50] Çalışkan G., Yıldırım G., Şenkardeş İ. (2020). A study on herbs used in food in Rize province within the context of gastronomy tourism. Int. J. Soc. Humanit. Sci. Res..

[51] De Giosa M., Tassi A.D., McDonald E.M., Ochoa R. (2021). First record of *Cenopalpus officinalis* Papaioannou-Souliotis (Tenuipalpidae) for Israel, Italy and Mexico and a redescription. Acarologia.

[52] Lawrence R.F. (1943). New South African mites of the genus *Tenuipalpus* Donnadieu (Tetranychidae). Trans. R. Soc. S. Afr..

[53] Meyer M.K.S. (1979). The Tenuipalpidae (Acari) of Africa; With Keys to the World Fauna.

